# IL-23 and PSMA-targeted duo-CAR T cells in Prostate Cancer Eradication in a preclinical model

**DOI:** 10.1186/s12967-019-02206-w

**Published:** 2020-01-14

**Authors:** Dawei Wang, Yuan Shao, Xiang Zhang, Guoliang Lu, Boke Liu

**Affiliations:** Department of Urology, Ruijin Hospital North, School of Medicine, Shanghai Jiao Tong University, No.999, Xiwang Road Jiading District, Shanghai, 201800 China

**Keywords:** PSMA, CAR T cells, IL23, Prostate cancer, IL-23, monoCAR, duoCAR

## Abstract

**Background:**

Prostate cancer is one of the most common adult malignancies in men, and nearly all patients with metastatic prostate cancer can develop and receive resistance to primary androgen deprivation therapy (ADT), a state known as metastatic castration-resistant prostate cancer (mCRPC). Recent reports demonstrated the great breakthroughs made by the chimeric antigen receptor T (CAR-T) cell therapy, which is significantly different from traditional T cells therapies. In spite of the progress of CAR-T technology in the treatment of lymphoma, leukemia, and other blood system tumor, there are still many difficulties in the treatment of solid tumors by CAR-T technology.

**Methods:**

In this report, we designed a panel of IL23mAb-PSMA-CARs, including PSMA-CAR, IL23mAb-T2A-PSMA-CAR, IL23mAb-PSMA-CAR, and PSMA-CAR (soluble IL23mAb). And we studied the function of these CARs in mice model.

**Results:**

Co-culture experiments with different CAR T cells have normal lysis function in vitro. The duo-CAR T cells co-expressing the IL-23mAb and PSMA-mAb had a significant higher population than the rest three different CAR T cells in co-culturing experiments at day 28, 35 and 42. A panel of cytokines were differentially secreted at higher amounts in IL23mAb-T2A-PSMA-CAR T cells than CAR T cells in other groups. In NOD/SCID IL-2 gamma (NSG) mice model, IL23mAb-T2A-PSMA-CAR T cells functioned significantly better than CAR T cells from the other groups and eradicated the tumor from these mice starting at day 14 post T cells injection and regained the body weight immediately. In IL23mAb-T2A-PSMA-CAR mice, CD45RO+ CD8+ T cells and CD127+ CD4+ CAR T cells were significantly increased. RNA sequencing revealed a difference expression pattern of genes in IL23mAb-T2A-PSMA-CAR mice. A reverse infusion experiment under the same model further proved the tumor eradication function of IL23mAb-T2A-PSMA-CAR T cells.

**Conclusions:**

We found that IL-23mAb combined PSMA CARs worked better than PSMA CAR only in Prostate Cancer Eradication, and we further discussed the mechanisms among different IL-23mAb combined PSMA CARs in Prostate Cancer Eradication.

## Background

Prostate cancer has become the most common solid tumor with high mortality in males in Europe and the USA, with less understanding of its pathogenesis and to be improved diagnosis approaches [1, 2]. Androgen deprivation therapy is effective for the treatment in early stage prostate cancer, however, it can lead the result that most of the patients develop castration-resistant prostate cancer (CRPC) [3, 4].The development of CRPC may be related to androgen receptor gene amplification, and the abnormally expression of regulatory factors of androgen receptors in prostate cancer. Currently, there is still no effective treatment for patients with CRPC. The genetic engineering of T cells is capable of introducing tumor-targeting properties to naturally occurring T cells, which can overcome the reliance on the endogenous immune system [5].

Given the fact that transduction with antigen-specific TCR can redirect T cell activity, the chimeric antigen receptor T cell (CAR-T) therapy has achieved a lot of success in treating cancers like leukemia, which may also provide a new way for the treatment of malignant solid tumors like prostate cancer [6–9]. Prostate-specific membrane antigen (PSMA) represents a suitable target for therapeutic purposes. Up to now, multiple ongoing clinical trials for prostate cancer CAR-T therapy based on PSMA-specific CARs have been reported. One is a Phase I trial of prostate-specific membrane antigen (PSMA)-targeted CAR-T in CRPC patients (NCT01140373) [10–12]. Another is a Phase I trial of PSMA-TGFβRDN CAR-T for CRPC (NCT03089203). The second trial is in purpose to evaluate the safety and feasibility of dual PSMA-specific/TGFβ-resistant, CAR-modified autologous T cells (CART-PSMA-TGFβRDN cells) in CRPC patients [13, 14].

The traditional CARs are generally composed of three sections, including extracellular antigen capturing section, transmembrane domain, and intracellular signal transduction part. The extracellular antigen capturing section is usually served by single-chain fragment variable (scFv) or domain antibody with the size much smaller than ScFv, to specific recognize and capture the surface antigens in tumor cells; the transmembrane domain consists of the transmembrane region of CD3, CD8, CD28, or FcεRI which can fix antigen capturing proteins on the surface of T cells to transduce the signal into the cells via the binding or recognition of the tumor cells; while the intracellular signal transduction section is composed of CD8, CD28, or CD137 intracellular area and CD3ζ, which contains the immune-receptor tyrosine-based activation motif (ITAM) [15–17]. Recently, more advanced generation of CAR-T was reported by introducing multiple costimulatory molecules or inducible costimulatory molecule, to further improve the tumor-killing abilities by enhancing T cell proliferation activity, cytotoxicity, and T cell survival rates. Some CARs even contains additional proinflammatory factor and co-stimulatory molecule ligands (4-1 BBL and CD40L) [13, 18–21].

TGF-b has been proved to induce metastasis and neoangiogenesis [22–26]. Expression of the dnTGF-bRII enhances antitumor immunity and T cell infiltration into tumors with potent antitumor responses. Results have been proved in the transgenic adenocarcinoma mouse prostate (TRAMP) mouse model of prostate cancer when utilizing this receptor [27]. Recent results also showed that dominant-Negative TGF-b Receptor enhances PSMA-Targeted Human CAR T Cell Proliferation And Augments Prostate Cancer Eradication [14]. Interleukin 23 (IL-23), which is a heterodimeric cytokine composed of an IL12B (IL-12p40) subunit and the IL23A (IL-23p19) subunit, is an inflammatory cytokine which plays a vital role in autoimmune diseases and in tumorigenesis [28]. Recent studies revealed that expression of the heterodimeric cytokine interleukin (IL)-23 is increased in human tumours, for IL-23 promotes inflammatory responses such as upregulation of the matrix metalloprotease MMP9, and increases angiogenesis but reduces CD8 T-cell infiltration [29]. IL-23 has also been proved of its tumor-promoting effect in mammary cancer mediated by infiltration of M2 macrophages and neutrophils in tumor microenvironment [30–34]. Recent study also showed that IL-23-induced immune cell activation aggravates gut inflammation and promotes growth of colon cancer [35]. In prostate cancer study, IL-23 produced by myeloid-derived suppressor cells (MDSCs) and can activate the androgen receptor pathway in promoting cell survival and proliferation. Results also showed that antibody-mediated depletion of IL-23 restored sensitivity to androgen-deprivation therapy in mouse model [32, 36]. All these studies highlighted the important role of IL-23 in tumor microenvironment.

Based on these knowledges, we designed a panel of IL23mAb-PSMA-CARs, including PSMA-CAR, IL23mAb-T2A-PSMA-CAR, IL23mAb-PSMA-CAR, and PSMA-CAR (soluble IL23mAb). These CARs were designed by a novel IL-23 specific antibody with higher affinity, combined with previous PSMA specific monoclonal antibody. And we found that IL-23mAb combined PSMA CARs worked better than PSMA CAR only in Prostate Cancer Eradication, and we further discussed the mechanisms among different IL-23mAb combined PSMA CARs in Prostate Cancer Eradication.

## Results

**Fig. 1:**
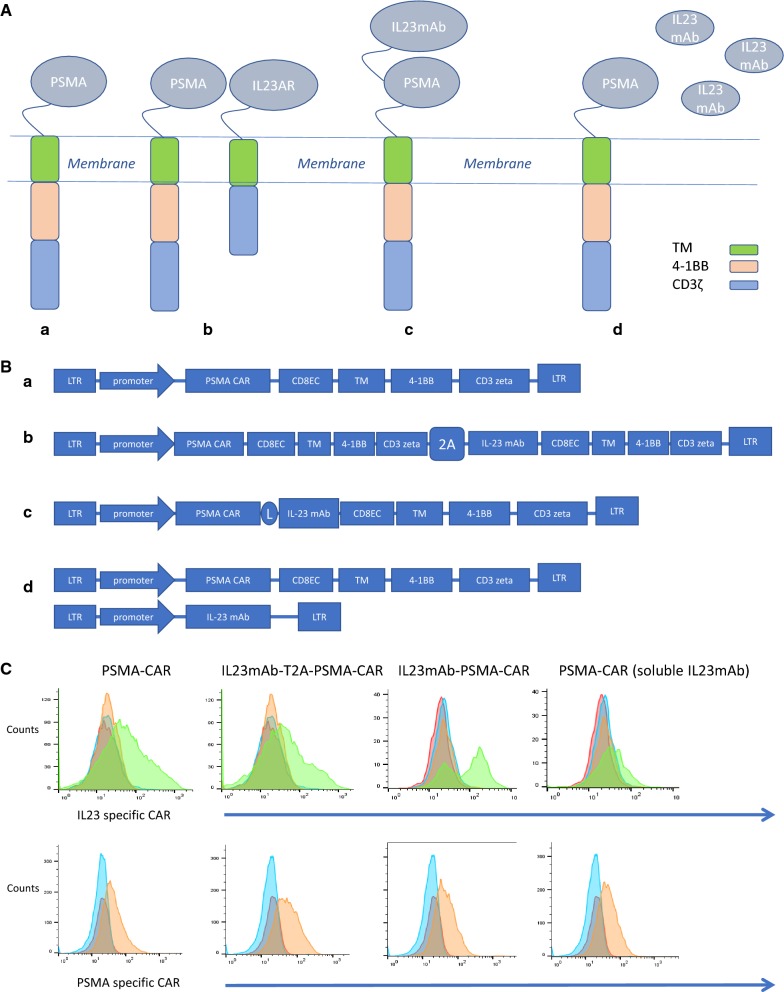

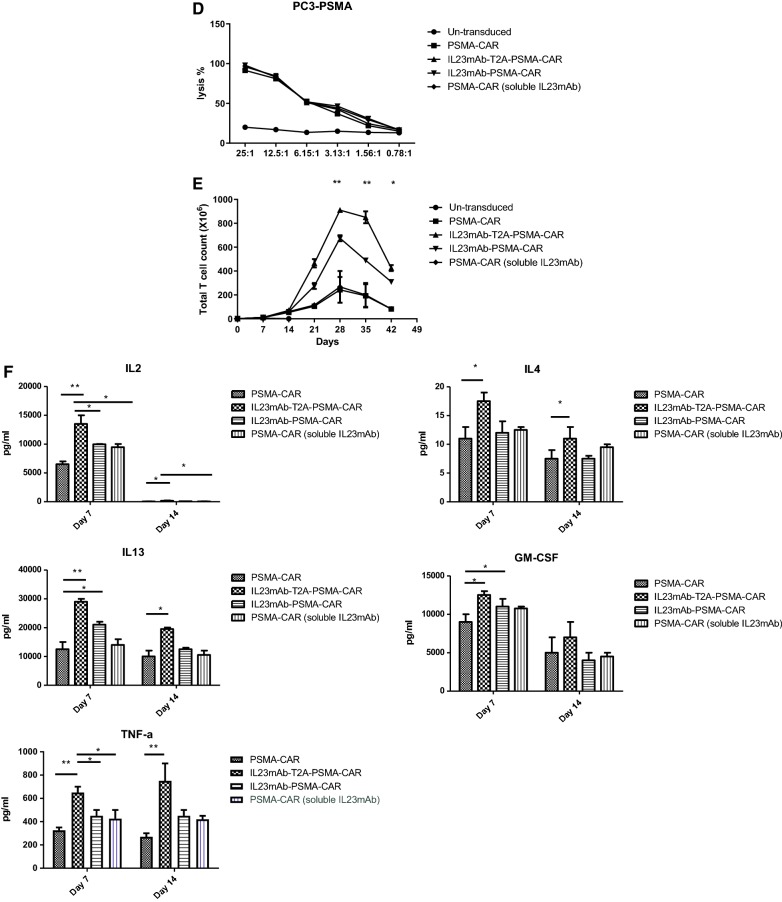
Illustration of the CAR architectures and functionality of CAR in vitro. **A** Cartoon illustration of each CAR used in the study: PSMA-CAR (a), IL23mAb-T2A-PSMA-CAR (b), IL23mAb-PSMA-CAR (c), and PSMA-CAR (soluble IL23mAb) (d). **B** Gene structure of CARs used in study: PSMA-CAR (a), IL23mAb-T2A-PSMA-CAR (b), IL23mAb-PSMA-CAR (c), and PSMA-CAR (soluble IL23mAb) (d). CD8 ectodomain (CD8EC), CD8 transmembrane domain (TM), 4-1BB costimulatory domain, and CD3ζ T cell signaling chain (CD3zeta). **C** Lentiviral transduction allows for efficient expression of different CARs in primary human T cells. In the upper lane, un-transduced cells (CAR negative cells) were labelled in red. Secondary antibody control was labelled in blue. Isotype control was in yellow and transduced cells were in green. In the under lane, un-transduced cells were labelled in red. Secondary antibody control was labelled in blue, and transduced cells were in yellow. **D** Specificity of lysis by different CAR T cells in PSMA + PC3 cells. **E** Antigen-specific proliferation of CAR T cells upon co-culture with PSMA + PC3 cells. **F** Cytokine analysis of different T cell supernatants. The error bars represent ± SD

The design of the CARs has been described in “Materials and methods” and Fig. 1A: all CARs used the 4-1BB costimulatory and CD3ζ-signaling domains by recruiting lentivirus vectors, with transduction efficiencies in primary human T cells in a range of 40–70%. Un-transduced cells were included as control. In the experimental groups of the PSMA CAR and IL23mAb (monoclonal antibody) combined PSMA CARs, the PSMA specific monoclonal antibody and IL-23 specific monoclonal antibody were screened from a 10^10^ phage display library. The library was constructed from human B cells. PSMA mAb has an EC50 of 20 nM in binding PSMA, and IL-23 mAb has an EC50 of 40 nM in binding human IL-23. Utilizing a T2A element or a linker element [37], we designed a panel of IL23mAb-PSMA-CAR, including PSMA-CAR, IL23mAb-T2A-PSMA-CAR, IL23mAb-PSMA-CAR, and PSMA-CAR (soluble IL23mAb) (Fig. 1A). PSMA-CAR is used as control. IL23mAb-T2A-PSMA-CAR is designed to express duo-CARs on the surface of engineered T cells. IL23mAb-PSMA-CAR is designed to express single CAR by linking PSMA-specific mAb and IL-23 specific mAb together. PSMA-CAR (soluble IL23mAb) is just a single PSMA CAR with expression of soluble IL-23 specific mAb. The design of structures of the CARs is depicted in Fig. 1B. Monospecific CAR construct was generated by fusing the PSMA specific mAb in-frame to a single-molecule CAR architecture that consists of a CD8 EC (ectodomain) followed by the CD8 TM (transmembrane) domain, the 4-1BB costimulatory domain to support CAR-T persistence, and a CD3ζ T cell signaling chain. DuoCARs (two-molecule CAR architecture) were generated in a monocarp (one-molecule CAR architecture) format by linking both CARs together using a 3xG4S motif (IL23mAb-PSMA-CAR) or using T2A element (IL23mAb-T2A-PSMA-CAR) (Fig. 1B).We obtained efficient co-expression of the PSMA-CAR with the IL-23-CAR. The expression of modified receptors in CAR T cells were examined and verified by Flow cytometry (Fig. 1C). These different PSMA CARs designed to induce specific lysis of human prostate cancer cells expressing PSMA. The PC3 prostate cancer cell line was recruited to construct a PSMA expressing PC3 cell line (described in “Materials and methods”). The PSMA positive PC3 prostate cancer cells were used for long-term co-culture experiments with different CAR T cells of each experimental group. All the engineered CAR T cells have normal lysis function in vitro (Fig. 1D). When these PC3-PSMA + prostate cancer cells were used for long-term co-culture experiments with different engineered CAR T cells, the duo-CAR T cells co-expressing the IL-23mAb and PSMA-mAb in T2A expression system had a significant higher population than the rest three different CAR T cells in co-culturing experiments at day 28, 35 and 42 (Fig. 1E), indicating the secretion of IL23 by PC3 (PSMA+) cells stimulate the growth or proliferation of IL23mAb-T2A-PSMA-CAR T cells. IL23mAb-PSMA-CAR T cells were also stimulated in proliferation, but not as much as IL23mAbT2A-PSMA-CAR T cells. Meanwhile, all four groups showed extended survival days than untransduced cells. At days 7 and 14, we harvested the supernatant for cytokine analysis, and we found that TH2 cytokines (IL4, and IL13, both p values are less than 0.05 in both time points) were differentially secreted at higher amounts in IL23mAb-T2A-PSMA-CAR T cells than CAR T cells in other groups. IL-2 secretion in IL23mAb-T2A-PSMA-CAR T cells was the highest among different CAR groups. In addition, significant increases were found in cytokines GM-CSF and TNFα in the IL23mAb-T2A-PSMA-CAR T cells compared to the PSMA-CAR T cells, IL23mAb-PSMA-CAR, and PSMA-CAR T cells with soluble IL23mAb (Fig. 1F).

**Fig. 2:**
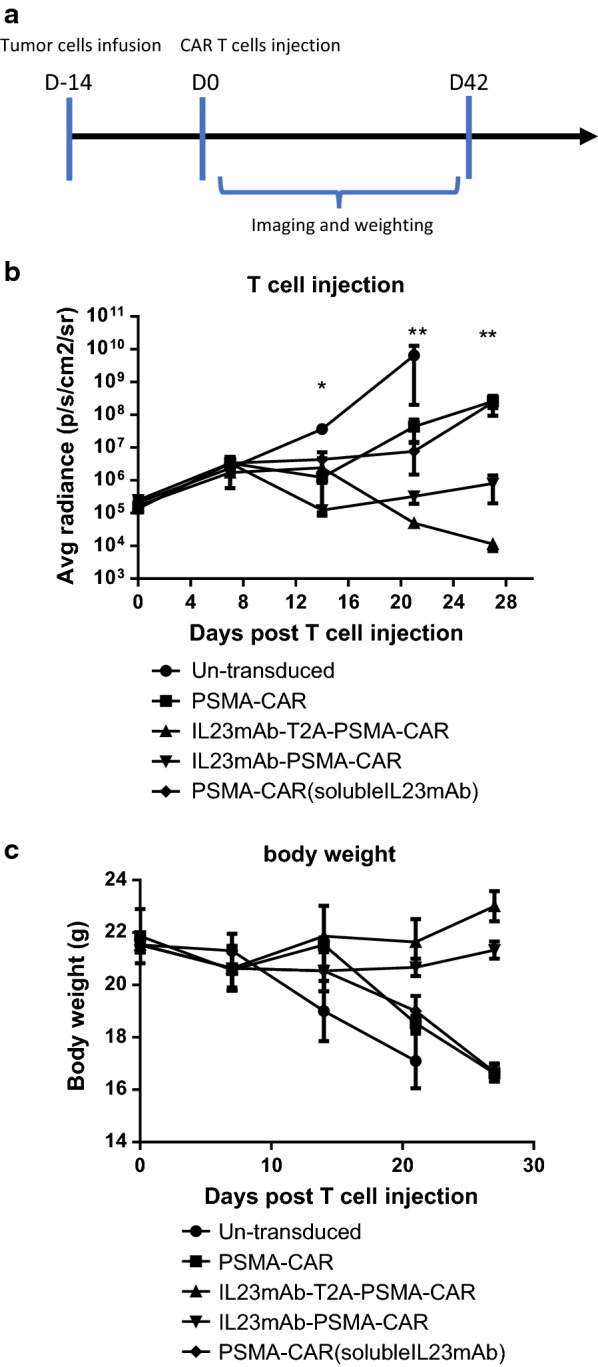
Different CAR T Cells in eradication of Prostate Cancer In Vivo. **a** Schematic of strategies in animal experiments. **b** T cell injection experiments. **c** Body weight measurement. The error bars represent ± SD

We further infused the PC3-PSMA human prostate cancer cells into NOD/SCID IL-2 gamma (NSG) mice, so that the tumor can be formed in mice. The PC3-PSMA cells were engineered to express luciferase for quantification. After 2 weeks of tumor forming, we infused 5 × 10^6^ CAR T cells intravenously (Fig. 2a). Results showed that the IL23mAb-T2A-PSMA-CAR T cells functioned significantly better than CAR T cells from the other groups and eradicated the tumor from these mice starting at day 14 post T cells injection and regained the body weight immediately (Fig. 2b, c). IL23mAb-PSMA-CAR T cells controlled the tumor growth until 28 days post T cell injection with regained body weight (Fig. 2c). PSMA-CAR T cells and PSMA-CAR T cells with soluble IL-23mAb can delay the tumor growth, but eventually failed to control them.

**Fig. 3:**
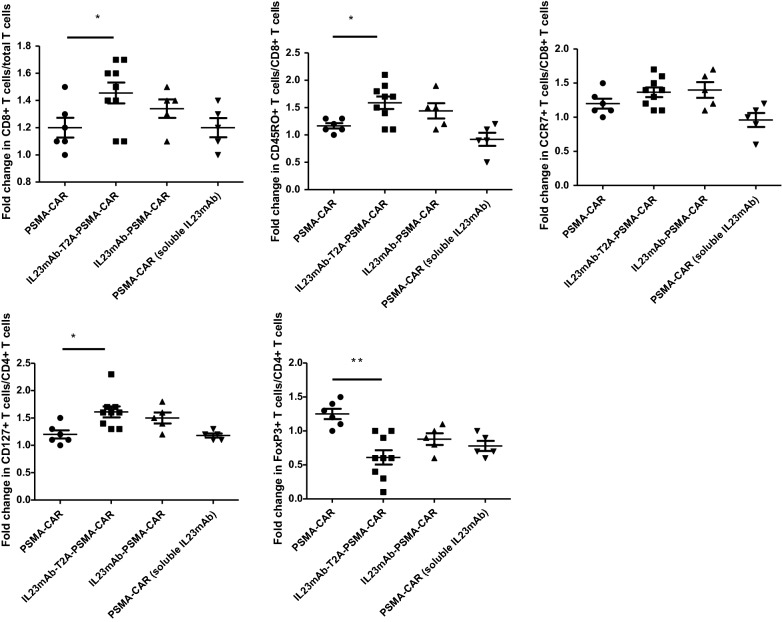
IL23mAb-T2A-PSMA-CAR T Cells was enhanced in cell proliferation

Given the observation that IL23mAb-T2A-PSMA-CAR T cells had a higher population than the rest of CAR T cells in co-culturing experiments, thus we have reason to suspect that IL23 secreted by tumor cells or existed in the micro-environment would stimulate the proliferation of the IL23mAb-T2A-PSMA-CAR T cells and IL23mAb-PSMA-CAR T cells. The higher detection of secretion of TH2 cytokines also indicated at least TH2 cells were enhanced in cell proliferation. In order to understand what type of T cells were proliferated in IL23mAb-T2A-PSMA-CAR T cells and IL23mAb-PSMA-CAR T cells, we sacrificed the mice at day 21 in all of these groups and isolated the PBMC for flow cytometry based analysis. We observed that the CD8+ T cell percentage was increased significantly in IL23mAbT2A-PSMA-CAR mice by day 21 (p = 0.0245). In addition, in IL23mAb-T2A-PSMA-CAR mice, CD45RO+ CD8+ T cells were increased (p = 0.0123), while CCR7+ CD8+ T cells are not significantly increased (p = 0.2312), in comparison to those in the rest of CAR groups. In CD4+ T cells, CD127+ CD4+ CAR T cells were increased to 1.2 folds (p = 0.0023), and FoxP3+ CD4+ T cells were decreased to 0.5 folds (p = 0.0003) (Fig. 3). Based on these observations, we briefly conclude that different CAR group has different flow cytometric profiling of CAR T cell subsets from PC3-PSMA co-culture. In order to further study the change of gene profiles of T cells in different CAR groups, we harvested total mRNA from these T cells from IL23mAb-PSMA-CAR group and PSMA-CAR group, and performed the longitudinal whole-transcriptome and microRNA microarray analysis. Results showed that there were 237 differentially expressed genes or microRNAs after 21 days infusion. Confirmed by cytokine analysis, IL-4, IL-5, and IL-13 were among the highest differentially expressed genes in the IL23AR-T2A-PSMA-CAR group (Table 1). The differentially expressed genes were mapped to the Kyoto Encyclopedia of Genes and Genomes (KEGG) pathways, and by using STRING analysis, we investigated enrichment for specific pathways. On day 21, more than 15 KEGG pathways were identified. The dominant pathways were mainly in cytokine interactions, STAT pathway, cell cycle, TCR signalling. Gene ontology (GO) analysis revealed similar pathways that were related to cell cycle and cell division (Table 1).

**Table 1:** Analysis of differentially expressed genes and pathways

	Top enhanced Gene Ontogeny pathway	Gene count
1	Mitotic cell cycle process	52
2	Cell cycle	59
3	Mitotic nuclear division	39
4	Cell division	22
5	Regulation of cell cycle	42
6	DNA replication	21
7	Small GTPase mediated signal transduction	29
8	Intracellular signal transduction	22
9	Positive regulation of mitotic cell cycle	13
10	Cytokinesis	10
11	Response to cytokine	12

	Top upregulated gene symbol	Fold change
1	IL13	10.1
2	IL4	5.9
3	IL5	3.5
4	IL4	3.1
5	EPAS1	2.9
6	IFNG	1.7
7	CSF2	1.5
8	PTGS2	1.5
9	ARG2	1.5
10	CCL3	1.5

	Top enhanced KEGG pathway description	Gene count
1	Cytokine‐cytokine receptor interaction	14
2	TNF signalling pathway	8
3	T cell receptor signalling pathway	5
4	Jak-STAT signalling pathway	7
5	Trancriptional misregulation	4
6	p53 signalling pathway	6
7	Cell cycle	7
8	Toll‐like receptor signaling pathway	7
9	Adipocytokine signaling pathway	5
10	Fc epsilon RI signaling pathway	5

**Fig. 4:**
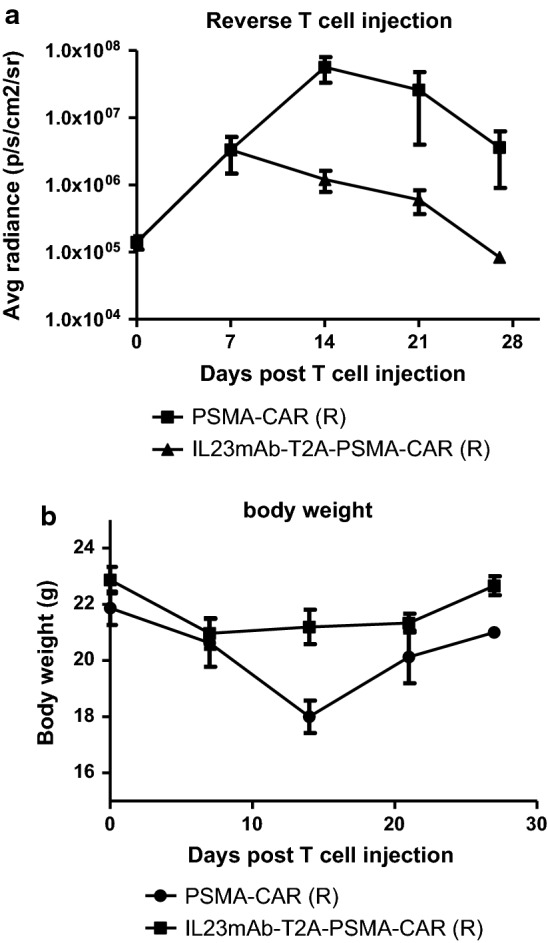
CAR T cells re-infusion in different CAR groups. **a** T cell injection. **b** Body weights

In order to further confirm the effects of the IL23mAb-T2A-PSMA-CAR T cells and PSMA-CAR T cells in tumor eradication, we performed a reverse infusion experiment under the same model (Fig. 2a). PBMC cells were collected from mice in IL23mAb-T2A-PSMA-CAR group at day 14 post T cells injection under the same model, and infused to mice in PSMA-CAR group at day 14 post T cells injection intravenously. In contrast, PBMC cells were collected from mice in PSMA-CAR group at day 14 post T cells injection under the same model, and infused to mice in IL23mAb-T2A-PSMA-CAR group at day 14 post T cells injection intravenously. We found that PSMA-CAR mice stared to regain the body weight and control the tumor after IL23mAb-T2A-PSMA-CAR T cells infusion (Fig. 4a, b).

## Discussion

In our study, we introduced a new panel of CARs, including duo-CAR by co-expressing engineered IL-23-specific CAR with PSMA-specific CAR, single CAR by linking IL-23-specific mAb and PSMA-specific mAb, and PSMA-CAR with soluble IL-23 mAb, and the results are encouraging. We are now evaluating the clinical utility of these CAR strategies, since all reported CAR T cell trials to date in patients against solid tumors are disappointed. IL-23 secreted by myeloid cells drives castration-resistant prostate cancer by activating the androgen receptor pathway, promoting cell survival and proliferation in androgen-deprived conditions. Hence, we designed a new strategy of CAR by introducing a IL-23 mAb either by duo-CAR or by linking to PSMA-CAR, in purpose of capturing the secreted IL23 by tumor cells or excited in the tumor environment. Through the comparison, IL23mAb-T2A-PSMA-CAR behaved more efficient in tumor eradication, and engineered T cells proliferation. In this CAR T cells infused mice, CD45RO+ CD8+ T cells, and CD127+ CD4+ cells were significant enhanced in proliferation, and Foxp3+ T cells were observed with reduced proliferation.

Under CD19-CAR and BCMA-CAR T cells treatment employed for hematologic malignancies in previous studies, CAR T cells proliferation or expansion were reported. Our observations are consist with previous reports. The enhanced expression of Th2 cytokines like IL-4 and IL-13 also indicates the potential CD4+ T cell proliferation or expansion. And these observations were confirmed by results of RNA sequencing. General sequencing results of T cell population after 21 days of infusion showed that there were at least 237 genes or microRNAs (miRNAs) that were differentially expressed. And genes in cytokine interactions, STAT pathway, cell cycle, and TCR signalling are alternated expressing in IL23mAb-T2A-PSMA-CAR T cells. These differences are current under understanding. The obvious thing is that all these pathways are proved related to cell proliferation and expansion.

In previous studies, CD4+ T cells and tumor cells engineered to secrete IL-4 have been shown to have potent antitumor effects [38, 39]. And different effects of engineered sub populations including CD4+ T cells, CD8+ T cells and Th17 cells have also been reported. Thus a CAR strategy that can enhance T cell abilities as well as T cell proliferation in a most efficient and specific T cell population and change the microenvironment in tumor at the meantime, will be more beneficial in solid tumor eradication. Our study provides knowledge in seeking good combinations of CARs with cytokines to achieve best tumor eradication and less side effects induced by cytokines. The potential usage of this IL23mAb-T2A-PSMA-CAR in human has been under investigation.

In our study, we found that the IL23mAb-T2A-PSMA-CAR strongly induced T cell activation, leading to increased cytokines production when compared to single-molecule CARs (IL23mAb-PSMA-CAR), although the binding of CAR types, independent of architecture, was quite similar. This observation strongly suggests that the higher cytokine secretion was due to the duoCAR architecture and not binding affinity of CAR. These results strongly suggest the importance of the architecture of the CAR, and such potent CAR-T cell effectors have the potential to increase the persistence in vivo in humans and maintenance of their effects.

## Materials and methods

### Vector design, CAR T Cell production and cell lines

IL23 specific antibody and PSMA specific antibody were isolated by phage display, and experiment was done by Y clone LLC and novel generation LLC. All CARs are designed basing on lentiviral vector. IL23mAb was first cloned into pELNS-tetR-T2A-Zeocin to replace the tetR cistron to form pELNS-IL23mAb-T2A-Zeocin. Next, the pELNS-IL23mAb-T2A-Zeocin cassette was cloned in the lentiviral expression plasmid from the Translational Research Program (pTRPE) lentiviral vector. A CAR was then designed and synthesized by Thermo Fisher Scientific. Lentiviral vectors were used to transduce human T cells from normal donor isolated from PBMCs by anti-CD4 and anti-CD8 microbeads (Miltenyi Biotec) and activated for 24 h with anti-CD3/CD28 Dynabeads (Thermo Fisher Scientific).The PC3 prostate cancer cell line (ATCC, CRL-1435) grow to confluency in vitro within 3–4 days of being seeded at 1 × 10^4^ cells/cm^2^ and cultured in D10 media consisting of DMEM with 10% FBS, HEPES, penicillin, and streptomycin. We used pTRPE lentiviral vector to introduce the PSMA protein and sorted the PMSA positive cells by BD FACS Aria III.

### Lentiviral vector production and CAR T cells production

Lentiviral supernatants were collected from tripartite-transfected HEK293T cells, concentrated by using ultracentrifugation, and used to transduce normal donor human T cells isolated from PBMCs by anti-CD4 and anti-CD8 microbeads (Miltenyi Biotec). Cells were activated for 24 h with anti-CD3/CD28 Dynabeads (Thermo Fisher Scientific). Transduced T cells were expanded for around 7–10 days in RPMI media (Gibco) with 10% fetal bovine serum (FBS), 4-(2-hydroxyethyl)-1 piperazineethanesulfonic acid (HEPES), penicillin, and streptomycin with 30–50 U/mL IL-2. Engineered CAR T cells were then stored in 90% FBS and 10% DMSO for future usage.

### Flow cytometry

LSRII (Becton–Dickinson) flow cytometer were used for analysis. Cells were stained in fluorescence-activated cell sorting (FACS) buffer (0.5% BSA Fraction V [Sigma] with biotin labelled first antibody, and streptavidin as secondary antibody.

### Tumor lysis assay and T cell proliferation assay

The Promega Luciferase Reporter Assay System (E1910) was used to measure percentage of cell lysis. After 16 h of co-culture of PC3 cells with T cells at effector:target ratios from 0 to 25:1, cells were harvested, lysed, and luminescence was measured (Promega system). In co-culture experiment, PC3 cells were irradiated with 15 Gy irradiation and plated at 2 × 10^5^ cells per well of 12-well plates. 24 h later, 10^6^ T cells total were plated with the irradiated PC3 cells in R10 media. In long term culture experiment, cell densities were over 1e6/mL after 5 days, and 1e6 T cells from these cultures were taken and re-seeded on freshly irradiated 0.2e6 PC3 cells. Cell numbers and size were evaluated using a Beckman Coulter Multisizer 3 Cell Counter. Cytokines in supernatant were measure kit (Life Technologies LHC6003M).

### Animal experiments

Institutional Animal Care and Use Committee in School of Medicine, Shanghai Jiao Tong University approved all animal experiments. Female NSG knockout (KO) mice were ordered from the Jackson Laboratory. 2 × 10^6^ PC3-PSMA cells were injected intravenously. Tumors were formed for 2 weeks, and CAR T cells were then injected. 3–4 representative mice were collected for bioluminescence imaging by Xenogen IVIS-200 Spectrum imaging system. Recombinant IL23 was injected following protocol intravenously. All experiments were performed under protocols in the Institutional Animal Care and Use Committee of the School of Medicine, Shanghai Jiao Tong University.

### Statistics

All statistical analysis was performed using GraphPad Prism v.5. For comparisons of 2 groups, 2-tailed un-paired t tests were used. *p  ≤ 0.05; **p ≤ 0.01;  ***p ≤ 0.001.

## Conclusions

IL-23mAb combined PSMA CARs worked better than PSMA CAR only in Prostate Cancer Eradication, and we further discussed the mechanisms among different IL-23mAb combined PSMA CARs in Prostate Cancer Eradication.

## Data Availability

The datasets used and/or analyzed during the current study are available from the corresponding author on reasonable request.
